# Abstracts and Gleanings

**Published:** 1885-02-20

**Authors:** 


### ABSTRACTS AND GLEANINGS.

**Climate of Las Vegas, N. Mex.**—Dr. F. H. Humphreys, who is sojourning at Hot Springs, Las Vegas, N. Mex., for his health, writes to the New York Medical Times:

I have looked into the matter of climatic cure for pulmonary complaints since I came here. The theory (and I am compelled to admit the force of it) is, first, the air in these high altitudes is purer, freer from destructive organic germs, and, though containing less oxygen than a lower level, is of greater oxidizing power, owing to the greater amount of that peculiar electrified form of oxygen, ozone. (See Tyndall's experiments in the Alps.)

Patients, on first coming here, find that they must inhale a greater quantity of air in order to obtain the usual amount of oxygen. This causes, at first, shortness of breath, and increased respiration, until the lung accommodates itself; the inspiration becomes deeper and every portion of the lung is receiving the highly oxidized air. The very effort to get the usual quantity of oxygen necessitates a greater amount of air inspired, and acts mechanically by distending the air cells to their utmost extent, in tearing open those partly occluded, in introducing into every portion of the lung dry, highly oxidizing ozone, which checks the exudation into the air cells and bronchia and breaks down and burns off the products already excluded. There is, therefore, an arrest and cure of the disease, providing the destruction of lung-tissue has not been too great, and that the patient has fair assimilative power and vitality.

The conditions, then, are, dryness, rarified air, and air of high oxidizing power. I think this place fulfills all these conditions.

At this great altitude, for the first two weeks, patients are apt to suffer somewhat, I have had quite an attack of catarrhal fever, with headache, coryza, sore throat, gastric and intestinal irritation. But almost all patients here claim that, after the first week or so, a great benefit is obtained by staying.

We can be out every day from 9 a. m. until 4 p. m. At noon the thermometer reaches as high as 70°. After 4 p. m. it rapidly grows cold; and at night goes away below freezing point, and we awake in the morning to find solid ice everywhere. We have had twelve days of sunshine thus far. Our days are like a mild October day at home. To-day (December 5, 1884) has been a trifle colder; thermometer only about 50°.

**The A. C. E. Mixture, Nitrous Oxide, and Ether.**—In one of the periodical discussions upon anæsthetics which recently took place before the London Medical Society, some more than ordinarily useful facts and conclusions were drawn out. They may be formulated as follows:

First. British surgeons are giving up the routine use of chloroform, except, perhaps, with children.

Second. The A. C. E. mixture was shown to be safer than chloroform, and almost as safe as ether, while more convenient. This mixture is composed of one part, by measure, of alcohol to two parts (measured) of chloroform and three of ether. This mixture should be made afresh just before being required for use. A drawback to its employment, though a small one, is that, in practice, it is found to act a little less rapidly than chloroform in the production of insensibility. It requires no complicated apparatus for its use, being doled out drop by drop on a napkin ; and, as an accumulating experience of many years in several metropolitan hospitals appears to testify, it is superior in point of safety for adult patients to chloroform alone. The exhilarating effects of the three parts of ether appear, in practice, to counterbalance the properties of the two parts of chloroform as a cardiac depressor.

One surgeon, Mr. Estes, uses chloroform for young children and the A. C. E. mixture for persons between the ages of six and eight and sixteen and eighteen.

Third. For safety, no anæsthetics equal ether and nitrous oxide.

Fourth. For both safety and rapidity, no other anæsthetics equal nitrous oxide gas and ether given together. The only objection to this combination appears to be connected with the apparatus that has to be employed. It is bulky, and demands a larger initial outlay than practitioners throughout the country, who seldom require to administer anæsthetics, would be disposed to incur. Still, for all who can employ them, nitrous oxide, used with ether, possess advantages not found in any other single anæsthetic agent, or combination of agents, at the present day known.—*N. Y. Med. Record.*

**Treatment of Chronic Dysentery by Voluminous Injections of Nitrate of Silver.**—At the meeting of the Clinical Society of London, held November 14, 1884, Dr. Stephen Mackenzie stated that extended experience had strenghtened his belief in the value of large enemas of nitrate of silver in the treatment of cases of chronic dysentery, or dysenteric diarrhœa. The mode of procedure he adopted was as follows :

The quantity of nitrate of silver to be used was dissolved in three pints of tepid water in a Leiter's irrigating funnel, which was connected by India rubber tubing with an æsophgeal tube with lateral openings. The patient was brought to the edge of the bed and made to lie on his left side, with his hips well raised by a hard pillow. The terminal tube, well oiled, was passed about eight inches into the rectum, and the fluid allowed to force its way into the bowel by gravitation. The injection rarely caused much pain, and often none. It usually promptly returned, but when long retained it was advisable to inject chloride of sodium to prevent absorption of the silver salt. Various strengths had been used, from 30 to 90 grains to three pints of water, but usually one drachm of nitrate of silver was employed. The treatment was based on the view, that whatever the nature of dysentery—whether constitutional or local in the first instance—the later effects were due to inflammation or ulceration of the colon, which was most effectually treated, as similar affections elsewhere, by topical measures. Sometimes one, sometimes two injections were required, and in some cases numerous injections were necessary ; but in all the cases thus treated—many of which had been unsuccessfully treated in other ways previously—the disease had been cured.

In most cases other treatment was suspended, but in some Dover's powder, or perchloride of iron. which had been previously administered, was continued or subsequently prescribed.

The cases narrated were :

1st. In which the disease had lasted several years, on and off ; two injections were used, and the case was cured in six weeks.

2d. Second attack, duration uncertain ; four injections used ; cured in five weeks.

3d. Duration, two months ; two injections used ; cured in three and one-half weeks.

4th. Duration, five years ; one injection used ; cured in three weeks.

5th. Duration, eighteen months ; two injections used ; cured of dysenteric symptoms, but remaining under treatment for diabetes.

6th. Duration, fourteen months ; one injection used ; cured in seven weeks.

The treatment, which laid no claim to novelty, was brought forward to elicit the experience of others who had tried it. or to induce others to employ it in suitable cases.—*Medical Press and Circular, 1884.*

**Muriate of Cocaine in Labor.**—Dr. J. R. Uhler : For some years I have been in the habit of using dilute solutions and ointments of carbolic acid, both as disinfectants during examinations, and to mitigate the pains of labor, but lately have thought that local anæsthesia can be more thoroughly induced by the employment of muriate of cocaine, either in solution, alone, or associated with dilute carbolic acid. A few days ago I had an opportunity of testing it upon a multipara, during the birth of her seventh child ; and, though the quantity of 2 per cent. solution employed was small, and the difficulties of keeping it *in situ*, owing to discharges, great, yet the results were satisfactory enough to encourage us to give it further clinical trial.

The case was not seen until the neck of the uterus was well dilated, nor was any of the drug purposely applied to the os, but at each examination after the discharge of the amniotic fluid, a few drops of the watery solution were smeared by the index finger around the labia and vagina, producing anæsthesia in spots, but more on the anterior than the posterior portions, probably because the drug in this situation, with the patient on her back, was not so readily washed away.

The uterine pains did not seem to be interferred with, but owing to anæsthesia of the vaginal walls, the voluntary straining efforts of the patient were not so prolonged as they had been in the other labor in which I had attended her, nor was the last pain severe enough to make her cry. Had the case been seen earlier, and the drug been used of greater strength. and more freely, or been applied in such a manner as to prevent its being washed away by the disoharges, a still better result would no doubt have been produced.

As it was, the case ended rapidly and very satisfactorily to both mother and child, and the former did not suffer from the after-soreness, which is such a common accompaniment of ordinary labor.—*Maryland Med. Journal.*

**True Croup.**—G. O. Smith. M.D., Odessa, New York, gives the following as the definition of True Croup:

An inflammation of the larynx trachea and, when extensive, the bronchi and its bifurcations, accompanied frequently by a formation of a false membrane. This disease is sometimes confounded with laryngo-tracheal diphtheria, but these two diseases are as distinct as the torrid and frigid zones. The one is a local inflammation only, the other a powerful zymotic poison of the blood accompanied by its peculiar local inflammation and exudation. There is no fetor in croup, but in diphtheria there is generally a horrible one. The nose of an expert is able to diagnosticate the two diseases. There are mild cases of croup that have but little tendency to the formation of a false membrane. In this case there will be the metallic ring in the cough, but there will be very slight, if any, febrile symptoms: but if the skin is hot, the child thirsty, and the temperature by the thermometer is steadily mounting up, then look out: there are breakers ahead. Fortunate for the patient if an experienced and intelligent physician is called in time, for here lies the only hope. Delay is death; prompt and skillful action is the patient's only salvation. As this disease most generally results from a sudden arrest of the secretion of the skin, let us, in any way we can, re-establish it. Give one or two drop doses of the tincture of aconite every hour, and a reliable tincture at that. Let no attenuated homœopathic nonentity be used. Put a fly blister from the chin to the sternum. To arrest the formation of a membrane already begun, give ten grains pulverized ipecac, of a good quality, and mix with it ten or fifteen grains of pure English calomel. If this treatment is given as soon as any rise of temperature begins the patient will recover; but if called too late, the physician, however skillful, may as well turn the patient over to the undertaker. If the medical man omits the calomel and death ensues, let him not console himself and the friends that all was done that conld be to save the patient's life, for such would not be true. When this disease involves the trachea and bronchi with a false membrane surgery is useless.—*Med. Summary.*

**Report of a Case of Labor in a Girl aged Eleven Years and Nine Months.**—T. H. Stallcup, M.D., at Texas Medical Association:

I was called February 2, 1882, to see Jennie M., who, I was informed, had been in labor about thirty-six hours, in the hands of a midwife. I hastened to the house of her father, who was known to me while an old slave, with some Indian blood, claiming to be a half-breed. Upon entering the house, and being astonished at the very youthful appearance of my patient, began inquiring of her father about her age, who informed me she had not arrived at her twelfth birth day. The next proceeding was to make a digital examination, which not only revealed the fact that she was pregnant but a well-formed occiput was plainly to be felt, descending the inferior strait, which was delivered three hours afterwards without the aid of instruments. Child, a girl, weighing a fraction less than seven pounds, with bruise on side of head and face; was asphixiated at birth, but was partially restored by the usual methods of blowing breath in the mouth and tepid bath, etc. After this, I made some inquiries as to the possible cause of the injury found upon the side of the face and head, and was informed that the day before labor set in she slipped off a gallery frozen over, striking her side against the end of the planks. I then left, to return later in the afternoon, finding the baby dead, and the mother resting quietly.

My object in reporting this case is to call the attention of the profession to the extreme youthfulness of the mother, and the injury to the brain in utero.—*Trans. Texas Med. Association.*

**Alleged "Vaccine" Against Yellow Fever.**—The Daily Times gives as news some account of the discoveries of Dr. Domingos Freire regarding the discovery of a yellow fever germ and yellow fever vaccine; and our contemporary wonders why the discovery does not attract more attention. Some of our medical contemporaries have also been heralding the discovery as something very new. The fact is, however, that Dr. Freire wrote a very full account of his work to the Sanitary News several months ago, and long before M. Bouley presented the subject to the Academie de Medicine. At an earlier time we gave an abstract of his conclusions, and added the opinion, which we still hold, that Dr. Freire has yet to win a reputation as a trustworthy observer. Some of his previous "discoveries" in bacteriology have turned out to be no discoveries at all.—*Ib.*

**Gastric Ulcer.**—In a case of gastric ulcer, Dr. O. B. Campbell, of St. Joseph, Mo. (in Clinical Record), says: I restricted her diet to skim milk, diluted with one-fourth lime water, from three to four ounces to be given every three hours, and gave a purge of calomel to relieve the portal circulation. I prescribed 5-drop doses of liquor potassii arsenitis, three times a day, and gave 20 grains of quinia during the twenty-four hours. My patient began to improve immediately; there was no more gastralgia nor vomiting; nor was there any appearance of blood in the stools. Six months after I began treating the case, all signs of disease had vanished. The cause of the ulcer was, I believe, portal congestion, caused by malaria.

**Treatment of Nasal Polypi.**—As a valuable contribution to the therapeutics of this unpleasant condition, we are glad to note that Dr. Richardson (in the Asclepiad) recommends the use of sodium ethylate in the treatment of nasal polypus. The caustic agent is applied by means of a probe made of soft cotton-wool, twisted into shape on the points of a pair of forceps. This cotton probe is saturated with the ethylate, and then plunged into the substance of the polypus. On removing the cotton, it commonly happens that the patient can expel the whole mass of destroyed polypus, in a semi-fluid form, by blowing the nose sharply. A second application ought to be made with a view of destroying the base of the polypus. The mode of action is said to be sufficiently clear. The ethylate is decomposed by contact with the water of the polypus into caustic soda and alcohol ; the latter coagulates the albuminoids, and the former acts as a powerful caustic. With the exception of some burning pain, no unpleasant effects seem to follow the use of this method.—*Medical Monthly.*

**The Treatment of Ringworm.**—Alder Smith, M. D. (British Medical Journal), gives the following: I desire now to call attention to a treatment for recent ringworm, where it does not extend over any large extent of surface. It is not a new remedy by any means, but. I believe, a new way of naming a well-known parasiticide. I have been trying. for some time to find out what vehicle penetrates most deeply in the hair follicles, and think it is chloroform. Chrysophanic acid is a very good parasiticide and, though it is insoluble in spirit and ether, yet it is soluble in chloroform. Chloroform also dissolves the fatty matter out of the hair follicles, and thus allows the parasiticide dissolved in it to penetrate deeply. During the last year I have used a solution of seven grains of the acid to the ounce of chloroform to all cases of recent ringworm, and believe it is the most efficient treatment I have yet tried. The small patches should be carefully marked out by cutting the hair very closely on them, and the chloroform solution should be well pressed and daubed into the places with a minute sponge-mop for five minutes, two or three times a day, according to the amount of irritation produced. The aim of the treatment is not to produce scabs, but to get the solution to penetrate deeply. The sponge-mop should not be much larger than a big pea, and should be continually dipped into the chloroform bottle, as the solution soon evaporates whilst it is pressed into the diseased spot, and leaves the yellow acid dry on the place. Great care must be taken that the solution does not run onto the forehead or into the eyes, and that the person using it does not inhale the vapor. I always give full directions about the care necessary in using such a potent remedy, and only employ it to weak places of the disease. It is well for the nurse to keep her face away from the sponge, and to use the chloroform in a current of air, and not in a small room. The places should be well washed every morning with hot water and soap, to remove any sebaceous matter or crusts, and the hair should be kept closely cut on them till the new hair appears, which is generally in about two or three months; but the remedy should be continued till all the diseased stumps have come out.—*Jour. Amer. Med. Assoc'n.*

**The Treatment of Corpulence on Physiological Principles.**—As analysed by the Birmingham Medical Review, Nov., 1884, Ebstein, in his work on corpulence, gives some valuable practical points for the reduction of obesity. According to him, fattening is strictly analogous to the fattening of cattle, and depends on overfeeding. He, however, disputes the current view that fat makes fat; on the contrary, he thinks fatty food protects the albumen and prevents its forming fat. His plan of treatment, therefore, consists in moderating the quantity of food, and while cutting off all vegetable carbo-hydrates, sugar, starch, etc., allowing a moderate quantity of fat, two or three ounces daily, to be taken. He also suggests that the diet should be monotonous, greasy and succulent, so as to cause satiety rapidly. He disallows beer, but permits light wines.

The plan advocated appears rational, and is free from the objection to Banting's method, which is too much like starvation. The following is the diet used successfully by Ebstein in one of his cases:

Breakfast: One large cup of black tea—about half a pint—without sugar; two ounces of white bread or brown bread, toasted, with plenty of butter.

Dinner: Soup, often with marrow; from four to six and one-half ounces of roast or boiled meat, vegetables in moderation, leguminous preferably, and cabbages. Turnips were almost, and potatoes altogether, excluded. After dinner, a little fresh fruit. For second course, a salad or stewed fruit without sugar. Two or three glasses of light wine, and immediately after dinner a large cup of black tea, without milk or sugar.

Supper: A large cup of black tea, as before. An egg, a little fat roast meat, or both, or some ham with its fat, bologna sausage, smoked or fried fish, about one ounce of white bread, well buttered, occasionally a small quantity of cheese, and some fresh fruit.

On this diet the patient lost twenty pounds in six months.

Ebstein insists on the necessity of always keeping to the restricted diet if the tendency to corpulence is to be successfully combatted.—*Ther. Gazette.*

**Lupus.**—A writer (Med. Times) treated successfully a number of cases of *lupus* as follows: Miss H., age 33, consulted me upon the subject of a small indolent ulcer, situated upon the left ala of the nose. Does not remember how it first appeared, but is quite sure that she has been troubled by it for more than three years. It has not increased in size for nearly a year. Her general health is good. The ulcer does not give rise to much pain. Occupying the edge and outer surface of the left nostril is a lupoid patch, the size of a sixpenny piece, presenting toward its upper aspect a smooth indolent ulcer, surrounded by thickened, congested and shiny skin.

Sulphurous acid, in the form of an oil, composed of ℥ j of Herring's alcoholic solution of sulphurous anhydride, with two ounces of castor oil, was applied. In ten days the ulcer had completely healed.

**Accidents Produced by Tincture of Arnica.**—A veterinary surgeon, M. Paul Cagny, calls attention in the Recueil de Medecine Veterinaire, to the severe symptoms which are often produced in horses, particularly in those with a delicate skin, by the free application of the tincture of arnica, and mentions the case of a foal which was kicked by another horse, and in which the slight abrasion so produced was treated by the prolonged local application of bandages soaked in pure arnica. The part became swollen and inflamed, and soon had all the appearances due to the action of a powerful vesicant.

This fact suggests that in human practice also numerous cases will be met with in which vesication or even erysipelas will follow the use of this application.

Tincture of arnica is too apt to be regarded as a panacea, particularly by the laity, for all bruises, etc., and even the physician is apt to regard it as a substance which can do no harm even if it does no good. But severe inflammatory action may be set up by its use, not only on wounds, but even on contusions where the skin is not broken. Its use should, therefore, never be left uncontrolled to the patient.—*Ther. Gazette.*

**On the Use of Iodoform in Erysipelas.**—C. Clark Burman, L.R.C.P. Edin., Belford, Northumberland, says: Since the introduction of iodoform by Dr. Glover, in 1837, its sphere of usefulness has been considerably enlarged. Its *internal* administration has, up to the present, been limited, being confined to some cases of phthisis, gastric ulcer, neuralgia and chronic rheumatic pains; but externally its use has become almost universal, either to the unbroken skin, or as a surgical dressing for wounds, ulcers and venereal sores. It is also largely employed in affections of the rectum, vagina, urethra, larynx, conjunctiva, and the oral and nasal cavities, where it can be readily applied. In fact, the topical effect of the drug determines to a large extent its employment, rather than its action upon the system; though, no doubt, by absorption it may influence the nervous centres, its chief action seems to be upon the peripheral terminations of sensory nerves.

Its action may be described as anæsthetic, absorbent, stimulant or resolvent, detergent or antiseptic, according to the conditions under which the application is made.

The purpose of this communication is to call attention to the well-marked property which the drug in question possesses of stopping the progress of erysipelas. I may say that I discovered this action accidentally, and as I am unaware of its employment in this affection I have been induced to publish the following cases in the hope of securing a more extensive trial for it in similar circumstances.

CASE I.—For some time I have been accustomed to use a solution of iodoform in collodion, of the strength of one ounce of iodoform to ten ounces of collodion, as an application to enlarged indurated glands in the neck and elsewhere. On one occasion, while painting some enlarged and painful inguinal glands in a man, the subject of chronic eczema of the lower extremities, he called my attention to a well-marked patch of erysipelas affecting the region of the left ankle and lower part of the leg. The usual characteristic appearances were present—considerable infiltration of the cutaneous textures, redness and heat, but no vesication; the patient complained of the burning heat rather than of actual pain. Bearing in mind Sir James Paget's experience ot collodion in erysipelas (see page 355 of Clinical Lectures and Essays), I painted the surface of the inflamed skin with the iodoform-collodion which I had just been using. On my next visit, to my surprise, I found the erysipelas was better; all heat and pain had gone, and the infiltration was rapidly subsiding. The glands in the groin were also much reduced in size and less painful, and in a few days all traces of the erysipelas had disappeared.

The observation of such a satisfactory result led me to adopt the same local treatment in my next case of erysipelas.

CASE 2.—A middle-aged woman had suffered from eczema of the scalp, which had given rise to an abscess over the occiput upon the left side. The abscess had opened spontaneously, and when I was called in had almost ceased discharging. Erysipelas had begun in the neighborhood of the abscess and spread along the left side of the head, involving the ear, and had then attacked the whole of the corresponding side of the face, extending beyond the middle line for a short distance along the forehead and implicating the right eye to a small extent. The swelling of the tissues upon the left side was so great as to close the eye completely; the pulse was full, rapid and hard: temperature over 101°, and during the previous night there had been some mental confusion and wandering. Owing to the intense burning pain experienced in the face and surrounding parts she had had no sleep for several nights. I painted the whole of the inflamed surface with the iodoform-collodion, going about half an inch beyond the line of redness. Almost immediately, she experienced relief from the pain, and remarked how very cooling the application was. That night she enjoyed several hours refreshing sleep. I prescribed:

℞ Liq. ferri perchlor ............................ ʒ vj

Sp. chloroformi ............................ ʒ ij

Infusi quassiæ ad ............................ ℥ viij.

M. ft. mist. Sig. ℥ j tertiis horis sumenda.

Upon my next visit a remarkable change had taken place. The swelling of the face had gone down to such an extent that the eye could be freely opened, all pain had ceased and the disease had not advanced beyond the original mark. Except for the stiffness caused by the collodion my patient felt nothing wrong. The application was repeated, and in a few days I put her on Easton's syrup, convalescence being fully established.—*Braithwaite's Ret.*

**Chronic Laryngeal Catarrh.**—Local applications are mainly to be relied upon in the treatment of this disease. These are applied to the larynx by the physician himself, usually by means of a brush and with the help of the laryngoscope. Various astringent remedies are employed for this purpose, and each physician usually has his favorite remedy. Ziemssen extols the use of strong solutions of nitrate of silver, sixteen grains and upward to the ounce, and they are, doubtless, amongst the most efficacious remedies: the good effect lasts much longer than that of milder astringents, so that an application once a week or once a fortnight will suffice. Others prefer the chloride of zinc (20 grains to the ounce), others chloride of iron (20 grains to the ounce), alum (10 grains to the ounce), tannin (10 grains to the ounce), and so on.

In the intervals of these applications the use of astringent or alkaline sprays may be beneficial. A Siegle's steam spray producer is the best apparatus for their production and application. As an astringent, a solution of tannin, 5 grains to the ounce, is perhaps the best. The spray should be applied twice a day for about five minutes each time. In cases where the mucous secretion is scanty and tenacious and difficult of removal from the mucous membrane and vocal chords, warm alkaline sprays are of much value. A solution of carbonate of soda and common salt, about 5 grains of each to the ounce, is one of the best.—*Medical Times and Gazette, July 12, 1884.*

**Hydrobromate of Conia in Epilepsy.**—The following cases are from a paper in Practitioner, January, 1884:

CASE 1.—A——, girl, aged eight: ill for two years with epileptiform seizures consisting of sudden flexions of the fore-arm (right), and a momentary vacantness of look. Latterly the attacks had become more severe, culminating in loss of consciousness. Hydrobromate of conia, in doses of half a grain three times a day, was prescribed. During the first week she had six slight "fits." The dose was then increased to five-eighths of a grain, and during the succeeding week she had no attack. The medicine was continued for four weeks, during which time she had no fits at all, and slept better. The drug was then discontinued for some weeks, when she returned for further treatment. During the administration of the drug, this patient complained of constant frontal headache.

CASE 2.—B——, male. aged twenty-two; suffered from true epileptic fits, with typical aura, convulsions, unconsciousness, and great headache afterward. One and a half grains of hydrobromate of conia was ordered twice a day. During the week this patient had nine fits. One and five eighths grains was now administered twice daily for a week. During this time the patient had four bad fits. He was now, at his own request, put under potassium bromide, ʒ j doses, three times a day, which kept them under.

CASE 3.—C——, female, aged thirty-four; had been ill for four years, with one or more fits every week, typically epileptic. While taking potassium bromide they were kept under. I ordered one grain of hydrobromate of conia twice a day to commence with. For a week she was better, with only one slight attack. The dose was increased to one and a quarter grains and during the next fortnight she had one slight fit. She was then ordered back to bromide.

CASE 4.—D——, girl, aged seven; has seven or eight fits a week of typical epileptic character. She has frequent right sided convulsions, the right arm being suddenly flexed. Sometimes these culminate in a real fit, with insensibility and rigidity. The child is an imbecile. As while under ʒ j doses of bromide the child still had frequent fits, I ordered one-quarter grain of hydrobromate of conia three times a day. For the first week she had five fits (all occurring the day after the medicine was changed). For the second week, there were seven fits. The drug was increased to half a grain three times daily. For a fortnight she was absolutely free from fits, and then had seven. The drug was continued for some weeks, but she still had fits occurring at irregular intervals, which were refractory to both conia and potassium bromide.

The drawbacks to the use of the drug appear in the complaints of headache, and where in large doses, of giddiness lasting for an hour after taking it, with sometimes a suffusion and congestion of the conjunctivæ.

**On the Therapeutic Uses of Resorcine.**—This drug, belonging to the aromatic series in chemistry, to which carbolic acid, salicylic acid, and many other drugs also belong, was discovered in 1860 by Hlassivetz and Barth, but has only recently come into use. Resorcine is very soluble in water, 95 parts in 100, and less so in ether, alcohol, glycerine and vaseline. The aqueous solution is neutral. It darkens on exposure to the air, is phosphorescent, and, treated with perchloride of iron, it gives a beautiful blue color; or, if sulphate of soda has been previously added, a deep garnet.

Its physiological effects have been well described by Justus Andeer. It destroys the low organisms, causing certain fermentations and putrefactions. A solution of 1 to 100 arrests alcoholic fermentation. A solution of 2·5 to 100 stops lactic fermentation, and is a valuable preservative liquid for anatomical specimens. It is, therefore, an antifervescent and antiputrescent of great power.

Justus Andeer and Peradon have experimented upon themselves, not only with small, but with large and dangerous doses. In doses of gr. xlv-lxxv a day, the drug only causes slight roaring in the ears, without other symptoms. ℈ viij, taken in twelve hours, produced dull pain in the head, with heaviness and loss of appetite. The same dose, taken in six hours, caused deafness, sighing respiration, vertigo and lassitude, and modified neither the temperature nor the pulse. Taken in two hours it caused a deep sleep, followed by a normal wakening. Taken in fifteen minutes it caused troubles of sight, hearing, odor and taste, followed by hallucinations. Peradon had the same experiences, with redness of the face, more or less profuse perspiration, and lowering of the temperature under large doses.

The therapeutic applications of resorcine are based on its antiseptic and antithermic properties, and it has been successfully used in certain infectious diseases. In typhoid fever it acts by combatting the infective principles and the high temperature of the disease.

### EXTERNAL USES.

On account of its antiseptic action, resorcine in solution may be used for dressing putrid or atonic wounds. It has the advantage over several other antiseptics of being odorless, though it is less astringent than carbolic acid. The solution is also useful as a topical application in syphilitic ulcerations; and, as shown by a recent pamphlet of Leblond and Fissiaux, it is as useful in the treatment of soft chancres as iodoform.

Resorcine has no irritating action on mucous membranes. For internal administration it is preferably given in an aromatic solution, or in sweetened water to which an aromatic syrup is added. The doses vary according to individual circumstances. For external use the vehicle may be water, or water with glycerine and alcohol. It can also be incorporated in pomades, etc.—*American Journal of Medical Sciences.*

**Cannibalism.**—The strange drama of cannibalism developed by the history of the survivors of the British yacht, Mignonette, has just come to an end in England. It will be remembered that the yacht foundered some months ago off the African coast, and the survivors, after a good many days of starvation and exposure, saw no hope of rescue, and, as a last resort, the captain killed a boy named Parker, and upon his flesh the party lived until help came. This is the barest outline of an awful story of suffering and starvation. The act was not deemed by the captain to be murder, but a measure of self-preservation, and was not concealed. A different view, however, was taken in England, and he was put upon trial for murder. The case attracted great attention from the unusual circumstances attending it, and much sympathy was felt for the accused.

The trial resulted in the jury finding the facts as above stated, but not determining whether they constituted murder or not. This was left to the judges, and the Court of Appeals recently decided unanimously that the act was murder. The sentence of death by hanging was then pronounced by Lord Coleridge, but the black cap was not prescribed, showing a desire to relieve the disgrace of the mode of death. It was also said that if clemency was to be exercised, it must be by the Queen. This has just happened, for the telegraph announces that the sentence has been commuted to six months' imprisonment without labor. This action by the Queen will be received gratefully by the community, for the deed, while technically a murder, had so many extenuating circumstances as almost to lift it out of the grade of crimes. It is a matter for rejoicing that the cases are exceedingly rare when the choice has to be made between death and the destruction of a fellow-creature.—*Med. and Surg. Rep.*

**The Oleomargarine War.**—The oleomargarine war waxes furious in some of the States, and in New York the last phase of the contest is a decision by the Supreme Court in the Second District that the law on the subject passed by the last Legislature is constitutional. There was one dissenting judge, and the case will be appealed, as the questions at issue are of a good deal of public importance. Much has been recently claimed for the "police power" of a State in the way of preventing trades or manufactures alleged to be injurious to the public, and it is important that some clear statement of the law should be made.

It is unquestionable that the manufacture of dirty or impure oleomargarine can be stopped legally, and also the offering for sale of oleomargarine for real butter; but the point to be absolutely settled is, whether all manufacture of the article can be prohibited in order to secure this objeet.

The progress of the case will be watched with some interest.—*Med. and Surg. Rep.*

**Indiscriminate Dosing.**—Medical Humbugs are not confined to the secret preparations so extravagantly advertised. It would be well if the law prevented prescribing by every one who is not a medical man. It is a popular notion that one who claims to teach some subjects, is capable of giving advise in others. We sometimes find clergymen prescribing for their parishoners, and editors going out of their sphere, if they have one, and advising their readers to use the same doses which they think they have found useful in their shattered health.—*American Agriculturist.*

**Treatment of Whooping Cough.**—Dr. J. Coopreider, of Taylorsville, Ind., writes to the Medical Summary that he has used the fluid extract of chestnut leaves for whooping cough with great success. He says: The dose employed is from 15 to 60 drops, according to age. If the child is old enough, I give it in hot water as an infusion, sweetened; to a small child, in simple syrup or elixir. It not only relieves or lightens the paroxysms, but will actually cure in from four to five days. I give from four to six doses per day, according to the severity of the case.

If good fresh leaves can be procured, I make the infusion as a tea, say two drachms of the leaves to half a pint of boiling water, and give two ounces at a dose, sweetened with white sugar.

**Cannabis Indica—Indian Hemp.**—Cannabis in small doses is one of our most potent remedies for the cure of subacute or chronic gonorrhœa; should be used only after the active inflammatory symptoms have been controlled by gelseminum, aconite, etc., and the urine rendered neutral and slightly alkaline by the proper use of saline diuretics. Many obstinate cases, which often perplex physicians and their patients, may be speedily corrected by this method without any local medication whatever. Painful urination, persistent burning in the urethra, are all corrected by cannabis. It is highly recommended in megrim or sick-headache, when the paroxysms are accompanied by gastric irritation; also, in hysteria, dysmenorrhœa, and uterine hemorrhage, especially when caused from neurotic excitement.—*Chicago Med. Times.*

**Cholera Infantum.**—Professor Bartholow says no single remedy is more efficient than brandy in cholera infantum. It must be given in sufficient doses (half drachm) frequently repeated. It may be administered in a little water without sugar.—*Col. and Clin. Record.*

**Cocaine in Laryngology.**—Dr. Edmund Jelinek contributes some interesting statements on this subject to the *Wiener Med. Blatt.* As the result of numerous experiments in Schrotter's clinic, he says that if the hydrochlorate of cocaine, either in powder or in the form of a strong solution, is applied to the mucous membrane, a marked diminution of the sensibility of the parts is noticed within a minute and a half. A solution of one part in ten or twenty is recommended, the formula suggested being as follows:

| | |
|---|---|
| Hydrochlorate of cocaine | 1 part |
| Alcohol | 2 parts |
| Distilled water | 3 parts. |

In examinations of the nose and larynx, it is sufficient to pencil the anterior and posterior surfaces of the soft palate, the posterior wall or the pharynx and the base of the tongue, the process being repeated after the lapse of a minute, if necessary. The local anæsthesia continues for about ten minutes. Prof. Schrotter speaks in the highest terms of the new remedy. He has produced most gratifying results by its use in cases of acute and tuberculous laryngitis attended with extreme irritation. Both Schrotter and Stork have removed papillomata from the vocal bands after inducing local anæsthesia, and testify to the value of the method.—*Med. and Surg Rep.*

**Sciatica.**—In the American Practitioner, Dr. Comiugor, of Indianapolis, recommends a somewhat new adaptation of an old treatment. The patient is placed under chloroform or ether; the affected limb is thoroughly flexed and extended, and made to move freely in all directions, and then at once put up in plaster of Paris, in which it is allowed to remain for a week. At the end of this time the cure should be complete. The treatment is best adapted for severe cases with contraction of the limb. It is virtually nerve-stretching without incision. With the forcible flexion we are already familiar, but not with the combination of a subsequent plaster case. We consider the method worth trying, and therefore record it.—*Edinburgh Med. Jour.*

**Chronic Bronchitis.**—Remember that opium is rarely ever admissible in the severe diffuse bronchial catarrhs of old persons and young children. When it is very necessary to secure a few hours' sleep, it is better to give from 5 to 20 grains of chloral, with an equal quantity of bromide of potassium or sodium.—*Medical Times and Gazette, July 12, '84.*

**A Reply to Koch.**—Professor Winckler, who claimed to have discovered true comma-bacilli of cholera in cholera morbus, has written an elaborate reply to Koch in the Gazette de Cologne. He denies that his methods of culture were faulty and reaffirms his original opinion, viz., that the comma-bacillus of Koch differs very little in form from that of cholera morbus, and that the differentia points laid down by Koch are not sufficient for diagnostic purposes.—*N. Y. Med. Jour.*

